# Nutritional Accuracy and Clinical Applicability of Large Language Model-Generated Therapeutic Diet Plans for Phenylketonuria: A Comparative Simulation Study

**DOI:** 10.3390/nu18193158

**Published:** 2026-09-25

**Authors:** Bahar Kulu, Banu Süzen, Nuriye Ece Mintaş, Sibel Burçak Şahin Uyar, Esra Özcan, Fulya Durğun, Neslihan Buse Tatlıdil, Nur Arslan, Volkan Hancı

**Affiliations:** 1 Division of Inherited Metabolic Disorders, University of Health Sciences, Tepecik Training and Research Hospital, İzmir 35170, Türkiye; 2 Department of Nutrition and Dietetics, School of Health Sciences, Cappadocia University, Nevşehir 50420, Türkiye; 3 Division of Inherited Metabolic Disorders, Muğla Training and Research Hospital, Muğla 48000, Türkiye; 4 Division of Inherited Metabolic Disorders, İzmir City Hospital, İzmir 35530, Türkiye; 5 Department of Nutrition and Dietetics, İzmir City Hospital, İzmir 35530, Türkiye; dyt.esraaa@gmail.com; 6 Department of Nutrition and Dietetics, Tepecik Training and Research Hospital, İzmir 35180, Türkiye; fulyadrgn@gmail.com (F.D.); b.tatlidil14@gmail.com (N.B.T.); 7 Division of Inherited Metabolic Disorders, Faculty of Medicine, Dokuz Eylül University, İzmir 35340, Türkiye; 8 Izmir Biomedicine and Genome Center (İBG), İzmir 35340, Türkiye; 9 Department of Anesthesiology and Reanimation, Division of Intensive Care, Faculty of Medicine, Dokuz Eylül University, İzmir 35340, Türkiye

**Keywords:** phenylketonuria, artificial intelligence, large language models, therapeutic diet, clinical nutrition, diet planning

## Abstract

**Background:** Large language models (LLMs) are increasingly used to generate nutrition-related recommendations, but their reliability for therapeutic diets requiring precise nutrient control remains uncertain. This study evaluated AI-generated one-day diet plans for standardized simulated cases of classical phenylketonuria (PKU). **Methods:** Eight standardized simulated cases representing infancy, adolescence, adulthood, and maternal PKU were evaluated. Between 8 and 10 February 2026, ChatGPT-5.2, Claude 4.5, and Gemini 3 were each used through their standard consumer-facing chat interfaces to generate one diet plan per case under two prompting conditions, yielding 48 plans. Nutrient composition was independently recalculated using BİAYS, and three blinded pediatric metabolic dietitians evaluated the plans using a structured 25-item checklist. **Results:** Across all 48 plans, phenylalanine provision calculated using BİAYS was below the case-specific prescription in 36 plans (75.0%). After Holm correction, none of the 12 paired AI-reported versus BİAYS-calculated nutrient comparisons remained statistically significant, while plan-level error metrics demonstrated substantial variability for several nutrients. Overall expert scores did not differ among models, whereas clinically specified structured prompts received higher expert ratings than limited-information zero-shot prompts. Inter-rater reliability was modest at the diet-plan level. **Conclusions:** AI-generated PKU diet plans may incorporate core dietary principles, but numerical plausibility does not ensure agreement with independent nutrient calculations or adherence to individualized clinical prescriptions. Independent nutrient verification and specialist metabolic-dietitian review remain necessary. Because only one output was generated for each case–model–prompt combination, the findings characterize the 48 evaluated outputs rather than within-model reproducibility.

## 1. Introduction

Phenylketonuria (PKU; OMIM 261600) is an inherited metabolic disorder characterized by accumulation of phenylalanine in the blood due to deficiency of the enzyme phenylalanine hydroxylase, which converts phenylalanine to tyrosine. If left untreated, elevated serum phenylalanine levels result in irreversible neurocognitive impairment [1]. Early diagnosis through newborn screening and lifelong dietary management have significantly improved clinical outcomes [2]. Dietary treatment remains the cornerstone of management; however, achieving optimal metabolic control remains challenging because PKU dietary therapy requires strict restriction of natural protein intake, individualized phenylalanine tolerance, and careful balancing of energy and nutrient requirements [2,3]. Given the lifelong nature of dietary management in PKU and the narrow therapeutic range of phenylalanine, even minor inaccuracies in dietary planning may have clinically significant consequences.

Dietary management in PKU is complex and dynamic, varying according to age, growth, metabolic control, and physiological conditions such as pregnancy [2,4]. Accurate meal planning requires adherence to established international guidelines and considerable clinical experience [2,5]. In clinical practice, dietitians play a central role in tailoring dietary prescriptions; however, providing individualized dietary management is time-consuming and requires regular adjustment throughout life.

In recent years, artificial intelligence (AI)-based large language models (LLMs) have emerged as promising tools in healthcare, with potential applications in clinical decision support, patient education, and personalized nutrition planning [6,7]. These models can rapidly generate dietary recommendations based on user-defined inputs. Recent studies have evaluated AI-generated meal plans in chronic diseases such as diabetes, obesity-related disorders, chronic kidney disease, and steatotic liver disease [8,9,10,11,12]. In some contexts, AI-generated diet plans have been shown to achieve comparable outputs to human experts in terms of general structure and macronutrient balance [6,13,14]. However, recent studies have also identified important limitations. AI-generated dietary recommendations may lack clinical accuracy, show variability across repeated outputs, and may not fully adhere to disease-specific guidelines, particularly in complex conditions requiring precise nutrient calculations [8,11,15]. Additionally, clinically relevant errors, especially in micronutrient content and disease-specific restrictions, have been reported, raising concerns about safety and reliability in real-world applications [11,15,16].

Recent studies have increasingly examined the use of large language models (LLMs) in nutrition and dietetics, including dietary assessment, personalized dietary recommendations, patient education, and meal planning [6,7,8,9,10,11,12,13,14,15,16]. However, much of this literature has focused on general dietary guidance or common chronic conditions. The application of generative AI to therapeutic diets for inherited metabolic disorders remains less well characterized. This distinction is particularly important in PKU, where dietary planning requires individualized control of phenylalanine intake, appropriate use of protein substitutes and specialized low-protein foods, adequate energy provision, and consideration of life-stage-specific nutritional requirements. Consequently, a diet plan that appears nutritionally plausible at a general level may not necessarily satisfy the clinical constraints required for PKU management.

Against this background, the present study evaluated AI-generated one-day therapeutic diet plans using standardized simulated cases of classical PKU representing infancy, adolescence, adulthood, and maternal PKU. Three LLMs were evaluated under two prompting conditions: a limited-information zero-shot condition and a clinically specified structured condition. The generated plans were assessed by comparing AI-reported nutrient values with independently calculated values, examining adherence to predefined case-specific energy and phenylalanine prescriptions, and obtaining blinded clinical evaluations from pediatric metabolic dietitians across four domains. Accordingly, the study aimed to characterize the numerical agreement, clinical target adherence, and expert-rated clinical suitability of the evaluated AI-generated PKU diet plans and to examine whether these outcomes differed according to AI model and the amount of clinical information provided in the prompt.

## 2. Materials and Methods

### 2.1. Study Design

This was a non-interventional, cross-sectional, simulation-based methodological evaluation of diet plans generated by large language models for classical phenylketonuria (PKU). No real patients, patient records, biological samples, clinical outcomes, or behavioral data were included. All case profiles were simulated, and all diet plans were generated specifically for this study.

The study evaluated the nutritional content, clinical appropriateness, and practical applicability of AI-generated PKU diet plans. Standardized simulated cases represented infancy, adolescence, adulthood, and maternal PKU. All models received the same case information and prompting instructions, and no AI-generated output was manually modified before nutrient analysis or expert evaluation.

The study was approved by the Non-Interventional Research Ethics Committee of SBÜ İzmir Tepecik Training and Research Hospital (decision number: 2026/01–25; date: 2 February 2026).

Reporting of this simulation-based LLM evaluation was informed by the Chatbot Assessment Reporting Tool (CHART) recommendations, where applicable to the present study design [17]. Because the study evaluated AI-generated therapeutic diet plans using standardized simulated clinical cases rather than an observational sample of human participants, STROBE was not used as the primary reporting framework.

### 2.2. Development of Simulated PKU Cases

Eight standardized clinical scenarios representing classical phenylketonuria (PKU) were purposively constructed according to current national and international PKU nutritional guidance. The scenario set included two infants (male and female), two adolescents (male and female), two adults (male and female), and two women with maternal PKU representing the first and third trimesters of pregnancy. The scenarios were designed as standardized test cases rather than as a statistically representative patient sample.

For each simulated case, age, sex, body weight, height, daily energy requirement, phenylalanine tolerance, and natural protein allowance were predefined according to the nutritional characteristics of the corresponding life stage. Daily energy requirements ranged from 1100 to 2600 kcal, phenylalanine tolerance from 200 to 750 mg/day, and natural protein allowance from 4 to 15 g/day (Table 1). These predefined nutritional targets were used consistently throughout the study. The eight scenarios were selected to provide coverage of clinically distinct PKU dietary contexts across major life stages. Two scenarios were included within each life-stage category. The number of scenarios was determined by this methodological coverage objective rather than by a conventional sample-size or power calculation. Because only two unique scenarios represented each life-stage category, life-stage findings were treated as descriptive and exploratory rather than as generalizable between-group comparisons.

### 2.3. Generation of Diet Plans Using Large Language Models

Three large language models (LLMs), ChatGPT-5.2, Claude Sonnet 4.5, and Gemini 3 Flash, were used to generate diet plans for each simulated PKU case. All three models were accessed through their standard consumer-facing chat interfaces rather than through an application programming interface (API), and all prompts were entered manually by the researcher. Diet plans were generated between 8 and 10 February 2026 in İzmir, Türkiye. No alternative model variant was manually selected; the default models available in the respective web interfaces at the time of data generation were used. No API-level generation parameters, including temperature, top-p, or seed, were manually specified or controlled by the researchers. No external plugins, file uploads, web-search tools, or retrieval-augmented generation systems were used.

For each simulated case, two prompting conditions were used. In the limited-information zero-shot condition, the model received basic demographic and clinical case information together with a general request to generate a complete one-day PKU diet plan; predefined nutrient targets and additional disease-specific dietary constraints were not explicitly provided. In the clinically specified structured condition, the prompt additionally included case-specific age, sex, body weight, energy requirement, phenylalanine tolerance, natural protein allowance, meal distribution, and relevant clinical dietary requirements. Thus, the two conditions differed in both prompt structure and the amount of explicit clinical information provided to the model. The study prompts were developed by two members of the research team (N.E.M. and B.S.) with expertise in clinical nutrition and PKU dietary management. The complete prompt templates and case-specific variables are provided in Supplementary Section S1.

All prompts were developed in English and applied consistently across the three AI models. One response was generated for each case–model–prompt combination; repeated independent generations were not performed. Each prompt was submitted in a new chat session, and each generated response was recorded without manual editing before nutrient analysis and blinded expert evaluation. The overall study workflow is presented in Figure 1.

### 2.4. Nutrient Analysis

Nutrient analyses were performed using the original AI-generated diet plans without manual modification. The nutrient composition of all 48 plans was independently recalculated using BİAYS (Besin İçerik Analiz Yazılım Sistemi), a cloud-based food-composition and dietary-analysis system developed in Türkiye [18]. According to the developer documentation, the BİAYS food-composition database incorporates the United States Department of Agriculture (USDA) including its 2024 data, the European Food Safety Authority (EFSA), and other scientifically accepted food-composition sources, while dietary reference values are based on the Dietary Guidelines for Türkiye (TÜBER) [19]. In the present study, BİAYS was used to independently recalculate energy, total protein, phenylalanine, and tyrosine values for each AI-generated diet plan.

Food quantities and portion descriptions were entered into BİAYS as reported in the original AI-generated diet plans. When an AI model reported a quantity as a range rather than a single value, the midpoint of the reported range was used for nutrient analysis. When a PKU-specific medical nutrition product or specialized low-protein food suggested by an AI model could not be directly identified or was not available in Türkiye, the closest locally available product was selected for independent nutrient calculation. For metabolic medical nutrition products, the closest appropriate product from Nutricia’s metabolic nutrition range available in Türkiye was used; for specialized low-protein foods, the closest corresponding Mayalıhane product was selected. Substitutions were based on similarity in product category and intended dietary use and were not assumed to be nutritionally identical to the products originally specified by the AI models. The number of individual product substitutions was not separately coded in the original dataset. The exact total number of food items contained in the continuously updated BİAYS database was not prospectively recorded; therefore, no unverified retrospective database-size estimate was reported.

Calculated values were interpreted with reference to the nutritional targets specified for each simulated case and current national and international PKU recommendations. Guidance data for the evaluation were derived from major international and national clinical references, including the European Guidelines on Diagnosis and Treatment of Phenylketonuria (2), Recommendations on Phenylketonuria in Turkey [20], the PKU Dietary Handbook [21], and Current State of the Treatment Landscape of Phenylketonuria [22]. The guidance data used in the evaluation are summarized in Table 2.

**Table 2:** Evidence-based clinical reference standards used for the development and evaluation of AI-generated PKU diet plans.

Category	Clinical Domain	Reference Standard Applied in This Study	Primary Source
Dietary prescription	Energy requirements	Age- and physiological status-specific energy requirements were determined according to current PKU dietary recommendations.	European PKU Guideline (2025) [2]; Turkish Guideline (2022) [20]
Dietary prescription	Phenylalanine prescription	Individual daily phenylalanine tolerance was defined according to age, metabolic control, and clinical status.	European PKU Guideline (2025) [2]; Turkish Guideline (2022) [20]
Dietary prescription	Natural protein allowance	Natural protein intake was individualized according to prescribed phenylalanine tolerance.	European PKU Guideline (2025) [2]; Turkish Guideline (2022) [20]
Diet composition	Protein substitute	Protein substitutes were incorporated to meet protein requirements while minimizing phenylalanine intake.	PKU Dietary Handbook [21]
Diet composition	Meal distribution	Daily phenylalanine intake was distributed across meals according to PKU dietary recommendations.	PKU Dietary Handbook [21]
Diet composition	Micronutrient adequacy	Vitamin and mineral requirements were considered through appropriate use of protein substitutes and low-protein foods.	PKU Dietary Handbook [21]
Clinical management	Blood phenylalanine control	Diet plans were evaluated for consistency with dietary recommendations aimed at maintaining therapeutic blood phenylalanine concentrations.	European PKU Guideline (2025) [2]; Current State of the Treatment Landscape of Phenylketonuria (2025) [22]
Clinical management	Life-stage adaptations	Dietary prescriptions were modified for infancy, adolescence, adulthood, and maternal PKU.	European PKU Guideline (2025) [2]; Turkish Guideline (2022) [20]
Nutritional assessment	Nutrient calculations	Nutrient composition of all diet plans was independently recalculated using BİAYS.	BİAYS developer documentation [18]

**Abbreviations**: PKU, phenylketonuria. The evidence-based reference standards summarized in this table were used both to develop the simulated PKU cases and to evaluate the nutritional adequacy, clinical appropriateness, and guideline adherence of AI-generated diet plans throughout the study.

### 2.5. Expert Evaluation

The 48 AI-generated diet plans were independently evaluated by three pediatric metabolic dietitians experienced in the nutritional management of phenylketonuria (PKU). Before evaluation, all diet plans were anonymized and assigned randomly generated identification codes. The reviewers were blinded to both the AI model (ChatGPT-5.2, Claude 4.5, and Gemini 3) and the prompting condition (limited-information zero-shot or clinically specified structured) used to generate each diet plan. All evaluations were performed independently, and the reviewers had no access to the ratings assigned by the other dietitians.

A structured 25-item expert evaluation checklist was developed specifically for this study to standardize the clinical assessment of AI-generated medical nutrition plans for PKU. The initial items were developed by a clinical nutrition researcher based on the clinical nutrition care process and key components of PKU dietary management. The checklist was subsequently reviewed by the pediatric metabolic dietitians involved in the expert evaluation, and its final form was established before the blinded assessment. The checklist was designed as a clinical assessment framework rather than as a psychometric scale. It comprised four clinically oriented domains: Clinical Appropriateness (7 items), Food Selection (10 items), Meal and Macro–Micronutrient Balance (4 items), and Applicability (4 items). Each item was rated on a five-point Likert-type scale ranging from 1 (strongly inappropriate) to 5 (fully appropriate). No separate pilot study or formal psychometric validation was performed.

For each diet plan and each dietitian, an overall checklist score was calculated as the mean of the 25 item ratings; domain-specific scores were similarly calculated within each domain. Higher scores represented more favorable expert ratings according to the predefined checklist criteria. The checklist scores were interpreted as structured expert judgments and not as measurements derived from a formally validated psychometric instrument.

The complete evaluation checklist and scoring structure are provided in Supplementary Section S2.

### 2.6. Statistical Analysis

Statistical analyses were performed using R software (version 4.6.1; R Foundation for Statistical Computing, Vienna, Austria). Data are presented as mean ± SD or median (Q1–Q3), as appropriate. Agreement/error analyses comparing AI-reported and BİAYS-calculated nutrient values used paired data. Mean bias was defined as the mean of the plan-level paired differences (AI-reported minus BİAYS-calculated value), rather than as a difference between group medians. Mean absolute error (MAE), mean absolute percentage error (MAPE), Bland–Altman bias and 95% limits of agreement, and paired tests were reported. Paired *t*-tests or Wilcoxon signed-rank tests were used, as appropriate to the distribution of the paired differences. These paired tests were used as supplementary assessments of systematic differences, while interpretation emphasized paired error and agreement metrics rather than normality testing alone. Holm adjustment was applied across the 12 paired nutrient comparisons (three AI models × four nutrients). Non-significant paired tests were not interpreted as evidence of agreement.

Clinical target adherence was evaluated using the independently calculated BİAYS values and the predefined case-specific prescriptions. For energy and phenylalanine, signed absolute and percentage deviations, MAE, MAPE, and the numbers of plans below and above the prescribed target were summarized overall and by AI model, prompting condition, and clinical scenario. No post hoc clinical acceptance margin was introduced because such a margin had not been prespecified in the study protocol. Natural protein and protein equivalent derived from phenylalanine-free/low-phenylalanine protein substitutes had not been recorded as separate variables; therefore, natural-protein target adherence and simultaneous attainment of all predefined targets could not be reliably assessed retrospectively.

To account for repeated use of the same eight simulated cases across all three AI models and both prompting conditions, BİAYS-calculated energy, protein, phenylalanine, and tyrosine values were analyzed using a two-factor within-case repeated-measures framework with AI model and prompting condition as within-case factors and the model-by-prompt interaction included. These analyses were considered secondary/exploratory, and Holm adjustment was applied across the 12 repeated-measures tests (four nutrients × three effects). Expert-evaluation analyses were conducted at the diet-plan level. AI-model comparisons used Friedman tests with case × prompting condition as matched blocks, and prompting-condition comparisons used paired Wilcoxon signed-rank tests across matched case × AI-model combinations. The overall expert score was treated as the primary expert-evaluation outcome; the four domain analyses were secondary/exploratory, with Holm adjustment applied separately to the four model-domain tests and the four prompt-domain tests. Inter-rater reliability was estimated from the 48 diet plans × 3 raters matrix using a two-way random-effects, absolute-agreement ICC, reported for both single measures [ICC(2,1)] and the average of three raters [ICC(2,k)], with percentile bootstrap 95% confidence intervals. All tests were two-sided, and *p* < 0.05 was considered statistically significant. Given the purposive use of eight standardized clinical scenarios and the methodological rather than population-inferential aim of the study, no formal a priori sample-size calculation was performed. Inferential analyses were interpreted cautiously and complemented by descriptive error and agreement metrics; statistical non-significance was not interpreted as evidence of equivalence.

## 3. Results

### 3.1. Characteristics of the Simulated PKU Cases

The eight simulated PKU cases covered infancy, adolescence, adulthood, and maternal PKU. Body weight ranged from 9 to 75 kg, target energy requirements from 1100 to 2600 kcal/day, phenylalanine tolerance from 200 to 750 mg/day, and natural protein allowance from 4 to 15 g/day. Case-specific characteristics and predefined nutritional targets are summarized in Table 1.

### 3.2. Nutrient Agreement and Error Analysis

A total of 48 AI-generated PKU diet plans (16 per AI model) were evaluated by comparing nutrient values reported by each AI model with values independently calculated using BİAYS. In addition to paired hypothesis testing, plan-level error was quantified using mean absolute error (MAE), mean absolute percentage error (MAPE), and Bland–Altman bias and 95% limits of agreement (Table 3).

For phenylalanine, MAE was 166.2 mg/day for ChatGPT, 144.1 mg/day for Claude, and 164.6 mg/day for Gemini, with corresponding MAPE values of 55.2%, 30.1%, and 53.0%. Although mean bias was relatively small for some model–nutrient comparisons, Bland–Altman analysis demonstrated wide limits of agreement at the individual diet-plan level. For phenylalanine, the 95% limits of agreement were −302.2 to 451.4 mg/day for ChatGPT, −346.4 to 367.2 mg/day for Claude, and −378.3 to 453.2 mg/day for Gemini.

After adjustment for multiple comparisons using the Holm procedure, none of the 12 paired AI-reported versus BİAYS-calculated nutrient comparisons remained statistically significant (all adjusted *p* > 0.05). Accordingly, the absence of a statistically significant paired difference was not interpreted as evidence of agreement.

Although the Gemini tyrosine comparison did not remain statistically significant after multiplicity correction (Holm-adjusted *p* = 0.131), the magnitude and dispersion of the discrepancy were notable: mean bias was +2303.8 mg/day, MAE was 2811.1 mg/day, MAPE was 207.1%, and the Bland–Altman 95% limits of agreement ranged from −3913.9 to +8521.4 mg/day. This was interpreted as a descriptive signal of substantial numerical error rather than as evidence of a statistically significant systematic overestimation.

### 3.3. Clinical Target Adherence of AI-Generated Diet Plans

Clinical target adherence was evaluated by comparing BİAYS-calculated energy and phenylalanine values with the predefined case-specific prescriptions (Table 4). Across all 48 diet plans, energy MAE was 278.8 kcal/day and energy MAPE was 16.6%. BİAYS-calculated energy intake was below the predefined target in 20 plans (41.7%) and above the target in 28 plans (58.3%).

For phenylalanine, overall MAE was 186.1 mg/day and MAPE was 36.8%. BİAYS-calculated phenylalanine intake was below the case-specific prescription in 36 of 48 plans (75.0%) and above the prescription in 12 plans (25.0%). Descriptively, phenylalanine MAPE was 41.8% for ChatGPT, 29.0% for Claude, and 39.5% for Gemini. The corresponding values were 36.9% for zero-shot prompting and 36.6% for structured prompting.

Case-specific descriptive analyses showed substantial heterogeneity. Phenylalanine provision was below the prescribed target in all six plans for Case 1, Case 5, and Case 8, whereas all six plans for Case 7 (maternal PKU, first trimester) were above the prescribed target. Case-specific signed deviations and MAPE values are provided in Supplementary Table S1.

Natural protein target adherence was not quantified because natural protein and protein equivalent from Phe-free/low-Phe protein substitutes were not recorded as separate variables in the original nutrient analysis.

### 3.4. Repeated-Measures Analysis of BİAYS-Calculated Nutrient Composition

In the within-case repeated-measures analysis, no statistically significant main effect of AI model was detected for energy [F(2,14) = 2.431, *p* = 0.124], protein [F(2,14) = 0.557, *p* = 0.585], phenylalanine [F(2,14) = 2.464, *p* = 0.121], or tyrosine [F(2,14) = 1.224, *p* = 0.324]. The distributions of BİAYS-calculated energy, protein, phenylalanine, and tyrosine values across the three AI models are illustrated in Figure 2.

Similarly, no statistically significant main effect of prompting condition was observed for energy [F(1,7) = 0.378, *p* = 0.558], protein [F(1,7) = 3.783, *p* = 0.093], phenylalanine [F(1,7) = 0.439, *p* = 0.529], or tyrosine [F(1,7) = 0.479, *p* = 0.511]. Model-by-prompt interactions were also not statistically significant for energy [F(2,14) = 0.280, *p* = 0.760], protein [F(2,14) = 0.585, *p* = 0.570], phenylalanine [F(2,14) = 0.015, *p* = 0.985], or tyrosine [F(2,14) = 3.232, *p* = 0.070]. None of the 12 tests remained statistically significant after Holm correction. Given the limited number of unique simulated cases (*n* = 8), these analyses were interpreted as exploratory, and statistical non-significance was not considered evidence of equivalence.

### 3.5. Expert Evaluation and Inter-Rater Reliability

Expert evaluations were reanalyzed at the diet-plan level to account for the clustered structure of the data. For each diet plan, an overall checklist score was calculated for each dietitian by averaging the 25 item ratings. These diet-plan-level scores were used for the expert-evaluation analyses. The mean overall expert scores were 3.225 for ChatGPT, 3.321 for Claude, and 3.252 for Gemini. In the matched analysis across case × prompting-condition blocks, overall expert scores did not differ among the three AI models (Friedman χ^2^ = 0.375, *p* = 0.829).

Plans generated under the clinically specified structured condition had a higher mean overall expert score than those generated under the limited-information zero-shot condition (3.337 vs. 3.195). In the paired analysis across the 24 case × model combinations, the difference was statistically significant (Wilcoxon W = 58.0, *p* = 0.015). This comparison reflects the combined difference in explicit clinical information and prompt structure and should not be interpreted as an isolated effect of prompt formatting.

In domain-level exploratory analyses, the prompting condition was associated with clinical appropriateness (raw *p* = 0.003; Holm-adjusted *p* = 0.013). The apparent AI-model difference in food selection did not remain statistically significant after Holm correction (raw *p* = 0.013; adjusted *p* = 0.053). No statistically significant model or prompting-condition differences were observed for the remaining domains after multiplicity adjustment (all adjusted *p* > 0.05). Life-stage scores were descriptive only: infancy, 3.471; adolescence, 3.062; adulthood, 3.191; and maternal PKU, 3.339. No inferential life-stage comparisons were performed because each category was represented by only two unique simulated cases.

Inter-rater reliability was recalculated using the 48 diet plans, rather than individual checklist items, as the unit of analysis. The two-way random-effects absolute-agreement ICC was 0.303 for single measures [ICC(2,1); bootstrap 95% CI 0.182–0.401] and 0.566 for the average of three raters [ICC(2,k); bootstrap 95% CI 0.407–0.673] (Table 5).

## 4. Discussion

This study evaluated 48 AI-generated therapeutic diet plans for standardized simulated cases with classical phenylketonuria using independent nutrient calculation and blinded expert assessment. The analyses distinguish three related but non-equivalent questions: whether AI-reported nutrient values agree with independent calculations, whether the independently calculated plans adhere to case-specific prescriptions, and how the plans are judged clinically by metabolic dietitians. After accounting for the repeated-measures structure, no statistically significant model, prompting-condition, or model-by-prompt effects were detected for BİAYS-calculated energy, protein, phenylalanine, or tyrosine; these non-significant findings should not be interpreted as evidence of equivalence.

Dietary management is the cornerstone of treatment for classical phenylketonuria, and accurate calculation of phenylalanine and other nutrients is essential. In the present study, the absence of statistically significant paired differences after multiplicity correction did not establish agreement between AI-reported and BİAYS-calculated values. Plan-level error metrics and Bland–Altman analyses demonstrated substantial variability for several nutrients, particularly phenylalanine and tyrosine. Moreover, comparison with the predefined case-specific prescriptions showed that 75% of plans provided BİAYS-calculated phenylalanine values below the prescribed target. These findings emphasize the distinction between numerical plausibility, agreement with an independent calculation, and adherence to an individualized clinical prescription.

A particularly notable finding concerned tyrosine estimation. Although the Gemini comparison did not remain statistically significant after correction for multiple testing, the magnitude and variability of the discrepancy were substantial, with a mean bias of +2303.8 mg/day, an MAE of 2811.1 mg/day, and an MAPE of 207.1%. This finding is relevant to the interpretation of AI-generated PKU diets because tyrosine has a distinct nutritional role in PKU, where endogenous conversion of phenylalanine to tyrosine is impaired and tyrosine is routinely supplied through phenylalanine-free amino acid substitutes. Importantly, however, a large discrepancy in estimated dietary tyrosine should not be interpreted directly as evidence of clinical toxicity or inappropriate plasma tyrosine concentrations. Current PKU guidance indicates uncertainty regarding specific tyrosine requirements, particularly in maternal PKU, and cautions against excessive supplementation in pregnancy because safety evidence is limited [2]. Therefore, the large numerical discrepancy observed here primarily highlights the need for independent verification of amino acid estimates in AI-generated therapeutic diets rather than demonstrating clinical harm.

Expert evaluation provided information not captured by nutrient calculations alone. Overall expert scores did not differ significantly among the three AI models, whereas plans generated under the clinically specified structured condition received higher overall expert ratings than those generated under the limited-information zero-shot condition. Domain-level analysis showed an association with clinical appropriateness after multiplicity adjustment. These findings should be interpreted as structured expert judgments rather than as scores from a validated psychometric instrument. Inter-rater agreement was lower when estimated at the appropriate diet-plan level: ICC(2,1) was 0.303 for an individual rater and ICC(2,k) was 0.566 for the mean of three raters. Thus, variability in clinical judgment should be considered when interpreting the expert-evaluation findings.

The higher expert ratings observed under the structured condition should be interpreted in the context of the information provided to the models. Structured prompts explicitly supplied case-specific energy requirements, phenylalanine tolerance, natural protein allowance, meal distribution, and other clinical constraints that were absent from the zero-shot condition. Therefore, the observed difference cannot be attributed solely to prompt structure or prompt engineering. Rather, the comparison reflects AI-generated planning when explicit clinical constraints are supplied versus planning from more limited case information [23,24]. Notably, the repeated-measures nutrient analysis did not detect statistically significant prompting-condition effects on BİAYS-calculated energy, protein, phenylalanine, or tyrosine, while expert ratings differed overall and in the Clinical Appropriateness domain. This distinction suggests that expert-perceived clinical suitability and calculated nutrient composition capture different aspects of AI-generated therapeutic diet plans.

Another important consideration is whether more advanced retrieval-supported approaches could improve the clinical grounding of generated diet plans. Recent studies have suggested that retrieval-augmented generation (RAG) may improve the factual accuracy and clinical relevance of AI-generated responses by integrating external, domain-specific knowledge sources [25]. RAG-based approaches have also shown potential in the rare-disease setting. Zelin et al. developed a knowledge-guided RAG approach for rare-disease diagnosis and demonstrated improved diagnostic performance compared with standard ChatGPT under some prompting conditions [26]. In nutrition and dietetics, a recent scoping review identified a limited number of studies using RAG or fine-tuning to develop domain-specific language models, highlighting that this remains an emerging area [27]. These examples were included to illustrate the broader potential of retrieval-supported LLM approaches rather than as direct evidence of effectiveness in PKU. Evidence from other clinical contexts should not be directly extrapolated to PKU, and future studies should evaluate whether retrieval from authoritative PKU-specific guidelines, food-composition resources, and metabolic product databases can improve the accuracy and clinical reliability of generated therapeutic diet plans. Incorporating geographic context may further improve practical applicability, although location information alone would not ensure clinical accuracy.

Several recent studies have evaluated the use of large language models in nutrition and dietetics across a range of applications. However, most have focused on healthy dietary patterns, obesity management, diabetes, or patient education rather than therapeutic diets for inherited metabolic disorders [28,29,30]. In addition, previous studies primarily evaluated nutrient adequacy or the quality of AI-generated information [11,15]. In contrast, our study combined standardized simulated cases, quantitative nutrient validation, blinded expert assessment, and inter-rater reliability within a single evaluation framework. This combined approach enabled us not only to assess the numerical accuracy of AI-generated diet plans but also to evaluate their clinical applicability and practical usefulness in PKU management. Previous work in hyperphenylalaninemia has mainly focused on machine-learning models for predicting dietary phenylalanine tolerance and supporting precision dietary management [31]. By contrast, our study evaluated large language models for generating complete therapeutic diet plans for PKU using both quantitative nutrient analysis and blinded clinical evaluation.

This study has several strengths. It used standardized simulated PKU scenarios, independent nutrient recalculation, blinded assessment by three pediatric metabolic dietitians, and a predefined structured checklist to evaluate complementary aspects of AI-generated therapeutic diet plans. The analyses also preserved the matched structure created by applying each model and prompting condition to the same cases and distinguished numerical agreement from adherence to the clinical prescription.

This study has several limitations. The eight standardized simulated scenarios were designed to provide coverage of clinically distinct PKU contexts rather than population-level representation, and only two unique cases represented each life-stage category; life-stage findings are therefore descriptive and may reflect case-specific characteristics. Only one AI output was generated for each case–model–prompt combination, so within-model stochastic variability and reproducibility across repeated generations could not be quantified. All models were accessed through standard consumer-facing chat interfaces rather than APIs; generation parameters such as temperature, top-p, and seed were not manually specified or controlled, and more granular model-variant, build, or snapshot identifiers were not prospectively recorded. Because commercial LLMs and their interfaces may be updated over time, identical prompts entered later may not reproduce the outputs evaluated here. Accordingly, the findings characterize the 48 outputs generated under the specified study conditions between 8 and 10 February 2026 rather than stable or reproducible performance of the underlying models. The two prompting conditions differed in both structure and the amount of clinical information supplied, preventing attribution of observed differences to prompt formatting alone. Natural protein and protein equivalent from phenylalanine-free/low-phenylalanine protein substitutes were not recorded separately, precluding reliable retrospective assessment of adherence to the prescribed natural protein allowance. BİAYS is a Türkiye-based food-composition and dietary-analysis system; differences in food-composition sources, product availability, and locally selected substitutes may limit direct comparability with studies using other databases. When an exact AI-specified medical nutrition or specialized low-protein product was unavailable, the closest locally available product was selected according to product category and intended use. Because substitutions were not separately coded and substituted products were not necessarily nutritionally identical, this procedure may have introduced variation in calculated nutrient values. The study did not include diet plans independently constructed by metabolic dietitians as an active comparator; therefore, the findings should not be interpreted as establishing the performance of LLM-generated plans relative to clinician-generated PKU diets. Future studies incorporating clinician-generated reference plans would allow for direct comparison of nutritional accuracy, clinical appropriateness, and practical applicability. The expert evaluation checklist was developed specifically for this study as a structured clinical review tool and was not subjected to formal psychometric validation or pilot testing. Inter-rater agreement was limited, particularly for individual-rater scores, and expert findings should therefore be interpreted with appropriate caution. Finally, only three LLMs and English-language prompts were evaluated, which may limit generalizability to other models and languages.

## 5. Conclusions

The evaluated AI-generated PKU diet plans incorporated several core principles of dietary management, but numerical plausibility did not consistently translate into agreement with independent calculations or adherence to case-specific prescriptions. Phenylalanine provision was below the prescribed target in 75% of the evaluated plans, while natural-protein adherence could not be assessed reliably from the recorded variables. After accounting for the repeated-measures structure, no statistically significant model, prompting-condition, or interaction effects were detected for BİAYS-calculated nutrient composition; these findings do not establish equivalence. Plans generated with explicit clinical constraints received higher expert ratings than those generated from limited information, but this difference cannot be attributed to prompt structure alone. Independent nutrient verification and specialist metabolic-dietitian review remain necessary before clinical use.

## Figures and Tables

**Figure 1:**
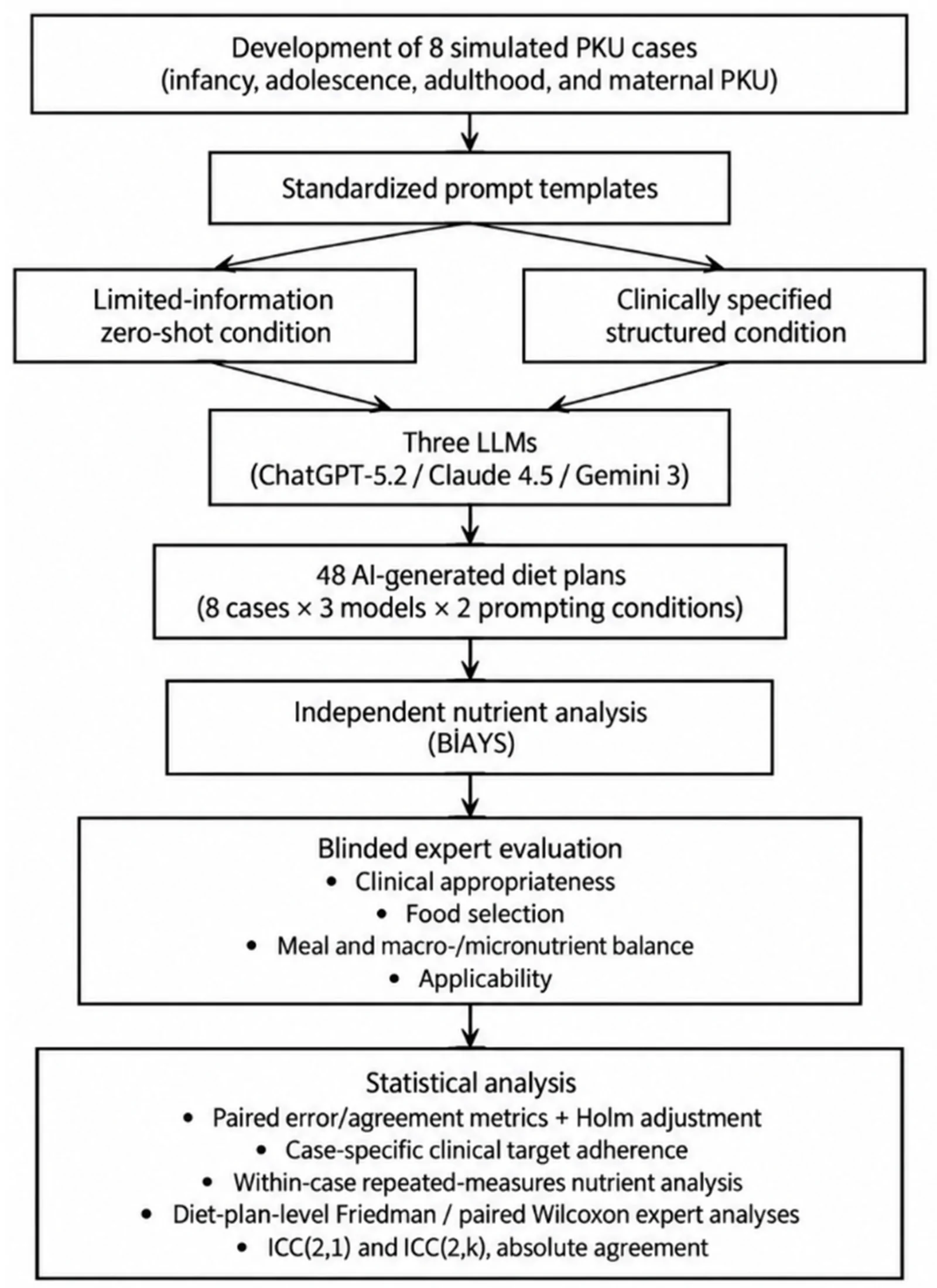
Study design and workflow of the evaluation of AI-generated diet plans for classical phenylketonuria.

**Figure 2:**
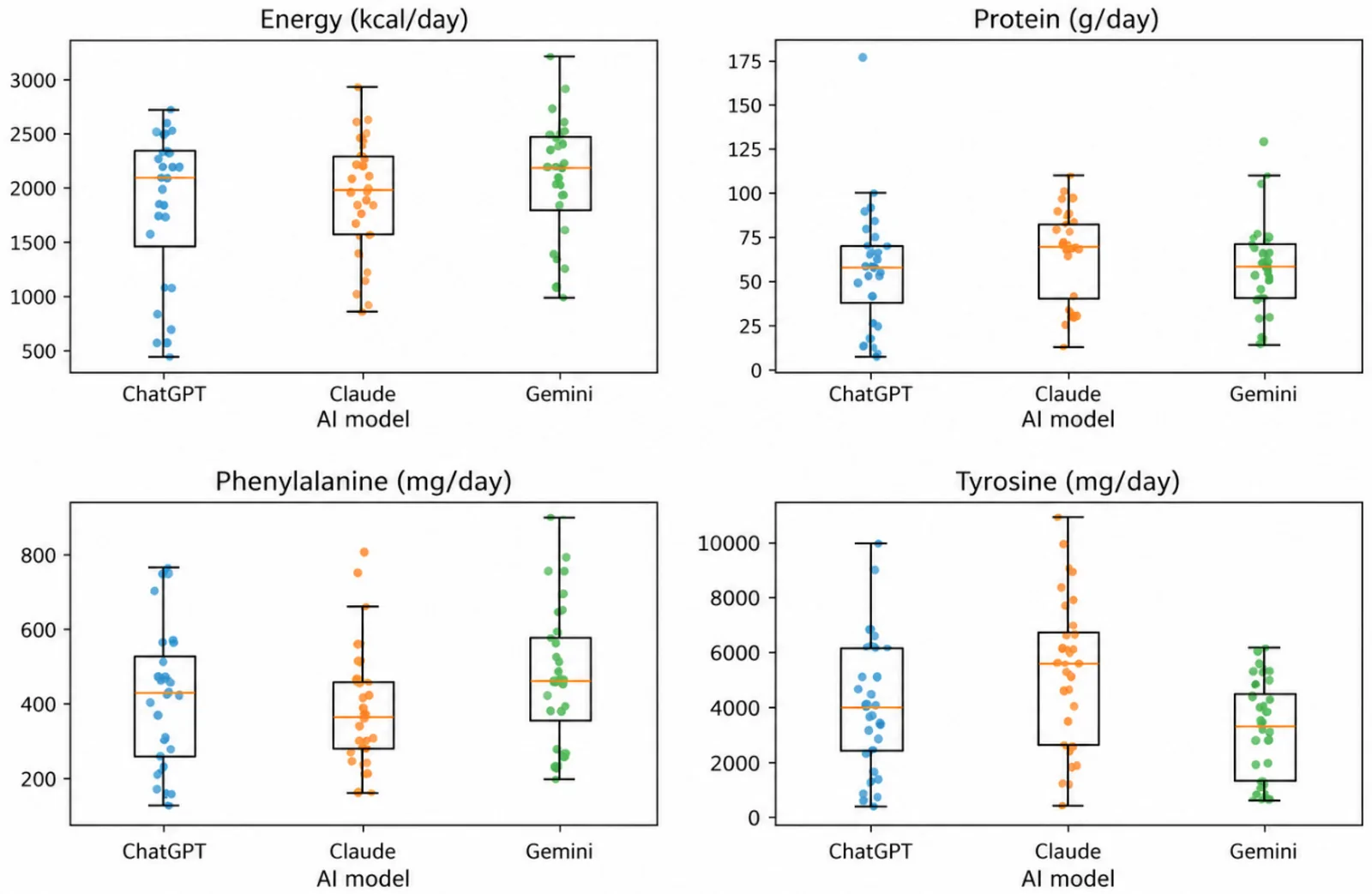
Distribution of BİAYS-calculated energy, protein, phenylalanine, and tyrosine values according to AI model.

**Table 1:** Characteristics and predefined nutritional targets for the simulated PKU cases.

Case	Clinical Scenario	Age (Years)	Sex/Status	Weight (kg)	Height (cm)	Target Energy (kcal/Day)	Phenylalanine Tolerance (mg/Day)	Natural Protein (g/Day)
1	Infant	1	Male	10	77	1100	250	5
2	Infant	1	Female	9	76	1100	200	4
3	Adolescent	13	Male	45	156	2200	550	11
4	Adolescent	13	Female	42	157	1850	500	10
5	Adult	22	Male	75	175	2600	750	15
6	Adult	22	Female	60	165	2100	450	9
7	Pregnancy (Maternal PKU)	25	Pregnant (1st trimester)	62	160	2200	400	8
8	Pregnancy (Maternal PKU)	25	Pregnant (3rd trimester)	68	160	2500	750	15

**Abbreviations:** PKU, phenylketonuria. Energy requirements, phenylalanine tolerance, and natural protein allowances were predefined according to the evidence-based clinical reference standards summarized in Table 2.

**Table 3:** Agreement and error between AI-reported and independently calculated nutrient values.

AI Model	Nutrient	AI-Reported Median	BİAYS-Calculated Median	Mean Bias †	MAE	MAPE (%)	Bland–Altman 95% LoA	Holm-Adjusted *p*
ChatGPT	Energy (kcal/day)	2100.0	2124.9	71.8	294.4	24.7	−632.2 to 775.7	1.000
	Protein (g/day)	62.5	55.3	−0.9	21.9	50.8	−66.7 to 65.0	1.000
	Phenylalanine (mg/day)	444.0	361.8	74.6	166.2	55.2	−302.2 to 451.4	1.000
	Tyrosine (mg/day)	3930.0	3787.7	138.5	2082.8	68.9	−5242.8 to 5519.8	1.000
Claude	Energy (kcal/day)	2150.5	2203.5	−196.4	327.3	14.6	−930.9 to 538.1	0.534
	Protein (g/day)	61.0	53.7	8.2	10.3	21.4	−24.9 to 41.2	0.369
	Phenylalanine (mg/day)	430.0	435.3	10.4	144.1	30.1	−346.4 to 367.2	1.000
	Tyrosine (mg/day)	3000.0	3165.0	−378.3	2024.8	82.0	−5336.2 to 4579.6	1.000
Gemini	Energy (kcal/day)	1900.0	1979.0	−92.0	221.0	12.6	−576.9 to 393.0	1.000
	Protein (g/day)	65.0	69.1	6.3	10.2	25.1	−24.4 to 36.9	1.000
	Phenylalanine (mg/day)	390.0	348.3	37.4	164.6	53.0	−378.3 to 453.2	1.000
	Tyrosine (mg/day)	5500.0	4245.0	2303.8	2811.1	207.1	−3913.9 to 8521.4	0.131

**Note**: Values are based on 16 paired diet plans per AI model. † Mean bias was calculated as AI-reported minus BİAYS-calculated values; positive values indicate overestimation and negative values indicate underestimation. MAE, mean absolute error; MAPE, mean absolute percentage error; LoA, limit of agreement. Holm adjustment was applied across the 12 paired comparisons. Statistical non-significance was not interpreted as evidence of agreement.

**Table 4:** Clinical target adherence of AI-generated PKU diet plans.

Group	*n*	Energy MAE	Energy MAPE (%)	Below Energy Target *n* (%)	Above Energy Target *n* (%)	Phe MAE	Phe MAPE (%)	Below Phe Target *n* (%)	Above Phe Target *n* (%)
AI model									
ChatGPT	16	326.7	20.8	9 (56.3)	7 (43.8)	200.1	41.8	13 (81.3)	3 (18.8)
Claude	16	311.4	17.2	3 (18.8)	13 (81.3)	159.5	29.0	9 (56.3)	7 (43.8)
Gemini	16	198.3	11.9	8 (50.0)	8 (50.0)	198.7	39.5	14 (87.5)	2 (12.5)
Prompting condition									
Zero-shot	24	261.4	16.2	11 (45.8)	13 (54.2)	191.0	36.9	16 (66.7)	8 (33.3)
Structured	24	296.2	17.1	9 (37.5)	15 (62.5)	181.2	36.6	20 (83.3)	4 (16.7)
Overall	48	278.8	16.6	20 (41.7)	28 (58.3)	186.1	36.8	36 (75.0)	12 (25.0)

**Note:** Clinical target adherence compares BİAYS-calculated values with the predefined case-specific energy requirements and phenylalanine prescriptions. MAE, mean absolute error; MAPE, mean absolute percentage error; Phe, phenylalanine. Below- and above-target classifications indicate direction of deviation from the exact predefined prescription and should not be interpreted as clinical inadequacy or adequacy because no arbitrary clinical acceptance margin was applied. Natural protein target adherence was not quantified because natural protein and protein equivalent were not recorded separately.

**Table 5:** Expert evaluation of AI-generated PKU diet plans using diet-plan-level analyses.

Outcome	Comparison/Group	Mean Score	Test Statistic	Raw *p*	Holm-Adjusted *p*
Overall expert score	ChatGPT	3.225	Friedman χ^2^ = 0.375	0.829	—
	Claude	3.321			
	Gemini	3.252			
Overall expert score	Zero-shot	3.195	Wilcoxon W = 58.0	0.015	—
	Structured	3.337			
Clinical appropriateness (A)	AI model	—	Friedman χ^2^ = 2.625	0.269	0.695
	Prompting condition	—	Wilcoxon W = 35.5	0.003	0.013
Food selection (B)	AI model	—	Friedman χ^2^ = 8.645	0.013	0.053
	Prompting condition	—	Wilcoxon W = 130.0	0.568	1.000
Meal and macro-/micronutrient balance (C)	AI model	—	Friedman χ^2^ = 2.926	0.232	0.695
	Prompting condition	—	Wilcoxon W = 88.0	0.128	0.384
Applicability (D)	AI model	—	Friedman χ^2^ = 0.237	0.888	0.888
	Prompting condition	—	Wilcoxon W = 118.5	0.794	1.000
Inter-rater reliability	ICC(2,1), single measures	0.303	Bootstrap 95% CI: 0.182–0.401	—	—
	ICC(2,k), average measures	0.566	Bootstrap 95% CI: 0.407–0.673	—	—

**Note**. For each diet plan, an overall checklist score was calculated for each dietitian by averaging the 25 item ratings. Diet-plan-level scores were used for inferential testing to avoid treating individual checklist items as independent observations. AI-model comparisons used Friedman tests with case × prompting condition as the blocking structure (16 matched blocks). Prompt comparisons used paired Wilcoxon signed-rank tests across 24 matched case × model pairs. Holm adjustment was applied separately across the four domain-level AI-model tests and across the four domain-level prompt tests. The overall expert score was treated as the primary expert-evaluation outcome and was not included in the domain-level multiplicity family. ICC(2,1) and ICC(2,k) were calculated from the 48 diet plans × 3 dietitians matrix after averaging each dietitian’s 25 item ratings within each plan; confidence intervals are percentile bootstrap 95% CIs based on resampling diet plans.

## Data Availability

The data supporting the findings of this study are available from the corresponding author upon reasonable request.

## Supplementary Materials

The following supporting information can be downloaded at: https://www.mdpi.com/article/10.3390/nu18193158/s1, Section S1: Standardized Prompt Templates Used for AI-generated PKU Diet Plans. Section S2: Expert Evaluation Checklist Used for Blinded Assessment of AI-Generated PKU Diet Plans. Table S1. Case-specific descriptive clinical target adherence of AI-generated PKU diet plans. Figure S1: Heatmap of item-level expert evaluation scores for AI-generated PKU diet plans.
