# Supplementary material for: Nutritional Accuracy and Clinical Applicability of Large Language Model-Generated Therapeutic Diet Plans for Phenylketonuria: A Comparative Simulation Study
Source: Nutrients. 2026 Sep 25;18(19):3158. doi: 10.3390/nu18193158 (PMC13667255; doi:10.3390/nu18193158)
Supplement: Supplementary file 1 [file nutrients-18-03158-s001.zip › Supplemantary Table S1.pdf]

Supplementary Table S1. Case-specific descriptive clinical target adherence of AI-generated PKU diet plans.

Clinical scenario	n	Energy median signed deviation (kcal/day)	Energy MAPE (%)	Phe median signed deviation (mg/day)	Phe MAPE (%)	Phe below / above target, n
Case 1 – Infant male	6	−3.0	31.24	−97.30	36.47	6 / 0
Case 2 – Infant female	6	−21.5	29.48	−67.35	29.52	5 / 1
Case 3 – Adolescent male	6	−204.89	24.24	−110.58	37.82	4 / 2
Case 4 – Adolescent female	6	+188.78	17.51	−98.99	35.39	4 / 2
Case 5 – Adult male	6	+75.0	8.00	−315.05	51.20	6 / 0
Case 6 – Adult female	6	+149.0	12.63	−94.56	23.84	5 / 1
Case 7 – Maternal PKU, first trimester	6	+58.0	5.86	+48.18	38.93	0 / 6
Case 8 – Maternal PKU, third trimester	6	−35.0	4.11	−340.31	40.89	6 / 0

Note: Signed deviation was calculated as BİAYS-calculated nutrient content minus the predefined case-specific clinical target. Positive values indicate values above the target and negative values indicate values below the target. MAPE, mean absolute percentage error; Phe, phenylalanine.
