# Supplementary material for: Nutritional Accuracy and Clinical Applicability of Large Language Model-Generated Therapeutic Diet Plans for Phenylketonuria: A Comparative Simulation Study
Source: Nutrients. 2026 Sep 25;18(19):3158. doi: 10.3390/nu18193158 (PMC13667255; doi:10.3390/nu18193158)
Supplement: Supplementary file 1 [file nutrients-18-03158-s001.zip › Supplementary Figure S1_Heatmap of item-level expert evaluation scores for AI-generated PKU diet plans.pdf]

## Heatmap of item-level expert evaluation scores for AI-generated PKU diet plans

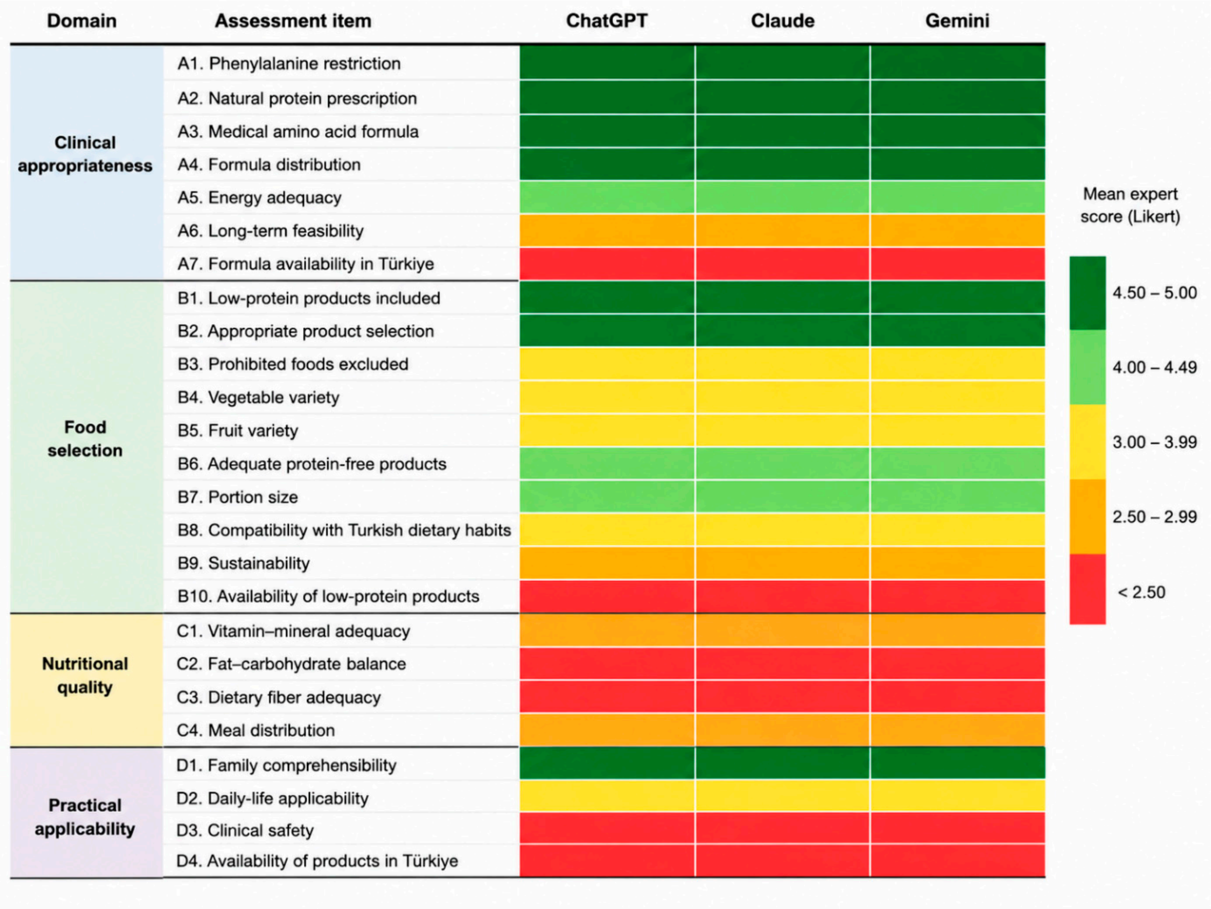


**Supplementary Figure S1**. The heatmap illustrates the mean expert evaluation scores assigned to each assessment item for PKU diet plans generated by ChatGPT-5.2, Claude 4.5, and Gemini 3. For visualization, assessment items are grouped into four abbreviated domain labels: clinical appropriateness, food selection, nutritional quality, and practical applicability. These labels correspond to the predefined checklist domains of Clinical Appropriateness, Food Selection, Meal and Macro–Micronutrient Balance, and Applicability, respectively. Color intensity represents the mean Likert score assigned by three independent pediatric metabolic dietitians, with score ranges indicated in the legend. Higher scores indicate greater expert-rated appropriateness.
