# Supplementary material for: Nutritional Accuracy and Clinical Applicability of Large Language Model-Generated Therapeutic Diet Plans for Phenylketonuria: A Comparative Simulation Study
Source: Nutrients. 2026 Sep 25;18(19):3158. doi: 10.3390/nu18193158 (PMC13667255; doi:10.3390/nu18193158)
Supplement: Supplementary file 1 [file nutrients-18-03158-s001.zip › Supplementary Section S1.pdf]

Supplementary Section S1.

Standardized Prompt Templates Used for AI-generated PKU Diet Plans

Section S1A. Standardized Zero-shot Prompt Template

The following standardized zero-shot prompt template was applied to all simulated PKU cases. Only the patient-specific demographic characteristics shown in Section S1C were modified for each case.

'*Prepare a complete one-day dietary menu for a patient diagnosed with classical phenylketonuria (PKU) using the patient characteristics provided below.'*

Patient characteristics

• Age: [AGE]

• Sex: [SEX]

• Weight: [WEIGHT]

• Height: [HEIGHT]

Present the dietary plan in structured tables reporting the following variables:

• Food items

• Food quantities

• Energy

• Phenylalanine

• Tyrosine

• Total protein

• Natural protein

Section S1B. Standardized Structured Prompt Template

The following standardized structured prompt template was applied to all simulated PKU cases. The same prompt structure was used throughout the study; only the patient-specific demographic and nutritional variables shown in Section S1C were modified.

Structured Prompt

'You are a clinical metabolic dietitian with expertise in the nutritional management of classical phenylketonuria (PKU).

Prepare an evidence-based one-day dietary plan according to current international and national PKU dietary guidelines using the patient characteristics and nutritional targets provided below'.

Patient characteristics

• Age: **[AGE]**

• Sex: **[SEX]**

• Weight: **[WEIGHT]**

• Height: **[HEIGHT]**

Nutritional targets

• Daily energy requirement: **[ENERGY] kcal/day**

• Phenylalanine tolerance: **[PHE] mg/day**

• Natural protein allowance: **[NATURAL PROTEIN] g/day**

Generate a complete one-day dietary plan including:

- phenylalanine-free medical formula,
- meal distribution,
- food quantities,
- nutrient calculations,
- complete daily menu,
- comparison of calculated and target nutrient values,
- and supporting guideline references.

The complete structured prompt contained additional instructions regarding guideline hierarchy, calculation rules, composite dish reconstruction, low-protein products, micronutrient assessment, output formatting, and reporting standards. These instructions remained identical for all simulated cases.

Output format

The response should be presented in structured tables with meal-specific nutrient values and daily nutrient summaries.

Section S1C. Case-specific Variables Inserted into the Standardized Prompt Templates

**Simulated case**	**Age**	**Sex**	**Weight (kg)**	**Height (cm)**	**Energy (kcal/day)**	**Phenylalanine tolerance (mg/day)**	**Natural protein allowance (g/day)**
Infant 1	1 year	Male	10	77	1100	250	5
Infant 2	1 year	Female	9	76	1100	200	4
Adolescent 1	13 years	Male	45	156	2200	550	11
Adolescent 2	13 years	Female	42	157	1850	500	10
Adult 1	22 years	Male	75	175	2600	750	15
Adult 2	22 years	Female	60	165	2100	450	9
Maternal PKU (first trimester)	25 years	Female	62	160	2200	400	8
Maternal PKU (third trimester)	25 years	Female	68	160	2500	750	15

Within each prompting condition, all prompt components remained identical across simulated cases except for the case-specific variables presented in Section S1C.

Note: Age, sex, weight, and height were provided in both prompting conditions. The predefined energy, phenylalanine, and natural protein targets were additionally provided only in the clinically specified structured condition.
