# Supplementary material for: Nutritional Accuracy and Clinical Applicability of Large Language Model-Generated Therapeutic Diet Plans for Phenylketonuria: A Comparative Simulation Study
Source: Nutrients. 2026 Sep 25;18(19):3158. doi: 10.3390/nu18193158 (PMC13667255; doi:10.3390/nu18193158)
Supplement: Supplementary file 1 [file nutrients-18-03158-s001.zip › Supplementary Section S2.pdf]

## Supplementary Section S2.

## Expert Evaluation Checklist Used for Blinded Assessment of AI-Generated PKU Diet Plans

The following checklist was independently completed by three pediatric metabolic dietitians for each AI-generated diet plan. The checklist was developed according to current PKU dietary guidelines and the Nutrition Care Process (NCP) framework of the Academy of Nutrition and Dietetics. Each item was scored using a five-point Likert scale.

| Score | Interpretation |
|---|---|
| 1 | Strongly inappropriate |
| 2 | Mostly inappropriate |
| 3 | Partially appropriate |
| 4 | Mostly appropriate |
| 5 | Fully appropriate |

### Reviewer Information

Diet code: ____________________

Case number: __________________

Reviewer code: ________________

Pregnancy: □ No □ Yes

### A. Clinical Appropriateness

| Item | 1 | 2 | 3 | 4 | 5 |
|---|---|---|---|---|---|
| A1. Phenylalanine restriction is appropriate | □ | □ | □ | □ | □ |
| A2. Natural protein allowance is appropriate | □ | □ | □ | □ | □ |
| A3. A phenylalanine-free amino acid formula is recommended | □ | □ | □ | □ | □ |
| A4. Medical formula is appropriately distributed across meals | □ | □ | □ | □ | □ |
| A5. Energy requirement is appropriate | □ | □ | □ | □ | □ |
| A6. Suitable for long-term dietary management | □ | □ | □ | □ | □ |
| A7. The recommended phenylalanine-free amino acid formula is available in Türkiye | □ | □ | □ | □ | □ |

### B. Food Selection

| Item | 1 | 2 | 3 | 4 | 5 |
|---|---|---|---|---|---|
| B1. Low-protein specialty products are included | □ | □ | □ | □ | □ |
| B2. Selection of low-protein products is appropriate | □ | □ | □ | □ | □ |
| B3. High-phenylalanine foods are appropriately excluded | □ | □ | □ | □ | □ |
| B4. Adequate vegetable variety is provided | □ | □ | □ | □ | □ |
| B5. Adequate fruit variety is provided | □ | □ | □ | □ | □ |
| B6. Sufficient low-protein foods are included | □ | □ | □ | □ | □ |
| B7. Portion sizes are age-appropriate | □ | □ | □ | □ | □ |
| B8. Food choices are compatible with Turkish dietary habits | □ | □ | □ | □ | □ |
| B9. Food choices are sustainable in daily practice | □ | □ | □ | □ | □ |
| B10. Recommended low-protein products are available in Türkiye | □ | □ | □ | □ | □ |

## C. Meal and Macro–Micronutrient Balance

| Item | 1 | 2 | 3 | 4 | 5 |
|---|---|---|---|---|---|
| C1. Vitamin and mineral adequacy is achieved | □ | □ | □ | □ | □ |
| C2. Fat and carbohydrate balance is appropriate | □ | □ | □ | □ | □ |
| C3. Dietary fiber intake is appropriate | □ | □ | □ | □ | □ |
| C4. Meal distribution is appropriate | □ | □ | □ | □ | □ |

## D. Applicability

| Item | 1 | 2 | 3 | 4 | 5 |
|---|---|---|---|---|---|
| D1. The meal plan is understandable for families | □ | □ | □ | □ | □ |
| D2. The meal plan is practical for daily use | □ | □ | □ | □ | □ |
| D3. The dietary plan is clinically safe | □ | □ | □ | □ | □ |
| D4. Recommended low-protein products are readily accessible in Türkiye | □ | □ | □ | □ | □ |

## Optional Reviewer Comments

______________________________________________________________

______________________________________________________________

Note: Domain-specific scores were calculated as the mean of the item scores within each domain. The overall expert score was calculated as the mean of all 25 checklist item scores.
